# In-situ forming dynamic covalently crosslinked nanofibers with one-pot closed-loop recyclability

**DOI:** 10.1038/s41467-023-36709-4

**Published:** 2023-03-02

**Authors:** Sheng Wang, Nannan Wang, Dan Kai, Bofan Li, Jing Wu, Jayven Chee Chuan YEO, Xiwei Xu, Jin Zhu, Xian Jun Loh, Nikos Hadjichristidis, Zibiao Li

**Affiliations:** 1 Institute of Sustainability for Chemicals, Energy and Environment (ISCE2), Agency for Science, Technology, and Research (A*STAR), Singapore, 627833 Singapore; 2 Institute of Materials Research and Engineering, Agency for Science, Technology, and Research (A*STAR), Singapore, 138634 Singapore; 3 Key Laboratory of Bio-Based Polymeric Materials Technology and Application of Zhejiang Province, Ningbo Institute of Materials Technology and Engineering, Chinese Academy of Sciences (CAS), Ningbo, 315201 P. R. China; 4 Polymer Synthesis Laboratory, Physical Sciences and Engineering Division, KAUST Catalysis Center, King Abdullah University of Science and Technology (KAUST), Thuwal, 23955 Saudi Arabia; 5 Department of Materials Science and Engineering, National University of Singapore, Singapore, 117576 Singapore

**Keywords:** Sustainability, Polymer synthesis, Synthesis and processing

## Abstract

Polymeric nanofibers are attractive nanomaterials owing to their high surface-area-to-volume ratio and superior flexibility. However, a difficult choice between durability and recyclability continues to hamper efforts to design new polymeric nanofibers. Herein, we integrate the concept of covalent adaptable networks (CANs) to produce a class of nanofibers ⎯ referred to dynamic covalently crosslinked nanofibers (DCCNFs) via electrospinning systems with viscosity modulation and in-situ crosslinking. The developed DCCNFs possess homogeneous morphology, flexibility, mechanical robustness, and creep resistance, as well as good thermal and solvent stability. Moreover, to solve the inevitable issues of performance degradation and crack of nanofibrous membranes, DCCNF membranes can be one-pot closed-loop recycled or welded through thermal-reversible Diels-Alder reaction. This study may unlock strategies to fabricate the next generation nanofibers with recyclable features and consistently high performance via dynamic covalent chemistry for intelligent and sustainable applications.

## Abstract (web summary)

Polymeric nanofibers are attractive nanomaterials owing to their high surfacearea- to-volume ratio and superior flexibility but designing durable and recyclable polymer nanofibers is challenging. Here, the authors integrate the concept of covalent adaptable networks to produce dynamic covalently crosslinked nanofibers via electrospinning.

## Introduction

Nanofiber is a class of nanomaterials with diameters ranging from tens to hundreds of nanometers with high surface-area-to-volume ratio and good flexibility, which makes them uniquely suitable for various applications, including energy harvesting/conversion/storage components, electronic/photonic devices, biomedical scaffolds, catalytic supports, “smart” mats as well as filtration membranes^1–4^. To date, natural/synthetic polymers, carbon-based/semiconducting/composite nanomaterials, and ceramics have been employed to fabricate nanofibers^5,6^. Among them, polymers are the most commonly used raw materials for nanofibers due to their wide range of sources and low cost^7,8^. Most thermoplastics with suitable molecular weight can be transformed into nanofibers by electrospinning as long as they can be dissolved in appropriate solvents with suitable viscosity or melted without degradation. However, compared to thermosets, thermoplastics have poor mechanical properties, dimensional solvent, and thermal stability^9,10^, limiting the applications of thermoplastics-based nanofibers. In contrast, thermosets-based nanofibers obtained by in-situ formation provide a powerful solution to these problems^11–15^, while thermosets are difficult to be recycled due to their highly crosslinked three-dimensional networks, resulting in a waste of resources and a huge burden on the environment^16–18^. Therefore, it is of urgent necessity to solve those issues and simultaneously endow the nanofibers with emerging functionalities and features.

Covalent adaptable networks (CANs) refer to a kind of crosslinked polymers with dynamic covalent bonds, which ideally combine the excellent properties of thermosets with the processability of thermoplastics^19–22^. In the conditions of use, CANs can remain stable, while rearrangement of the topological network or polymer chain depolymerization could occur when the dynamic bond exchange is activated by external stimuli^23,24^. Due to this unique feature, CANs have been extensively explored in the last decade, in synthetic and dynamic chemistry and materials science^25–31^. Moreover, when it comes to nanofibers, CANs are promising candidates for nanofibers considering the following advantages: (i) In the dynamic crosslinking system, the viscosity can be easily regulated by the pre-reaction between the crosslinking points^32–34^. It is promising to obtain nanofibers from low molecular weight polymers or low viscosity solution systems through the in-situ forming of dynamic crosslinks during electrospinning; (ii) The integration of crosslinking points can give nanofibers with excellent mechanical properties, dimensional, thermal, and solvent stability. (iii) The dynamic nature of CANs can endow the nanofibers with closed-loop recyclability, repairability and stimulus responsiveness, which are promising to solve the issue of limited lifecycle of nanofibers and expand the functions and applications of nanofibers. (iv) CANs based on various polymers and dynamic bonds/reactions have been developed^35^ and the diverse CANs can greatly enrich the family of nanofibers. Therefore, by integrating the dynamic covalent chemistry, nanofibers are able to be developed with new dimensions of simplicity, practicality, smart and sustainability.

**Fig. 1:**
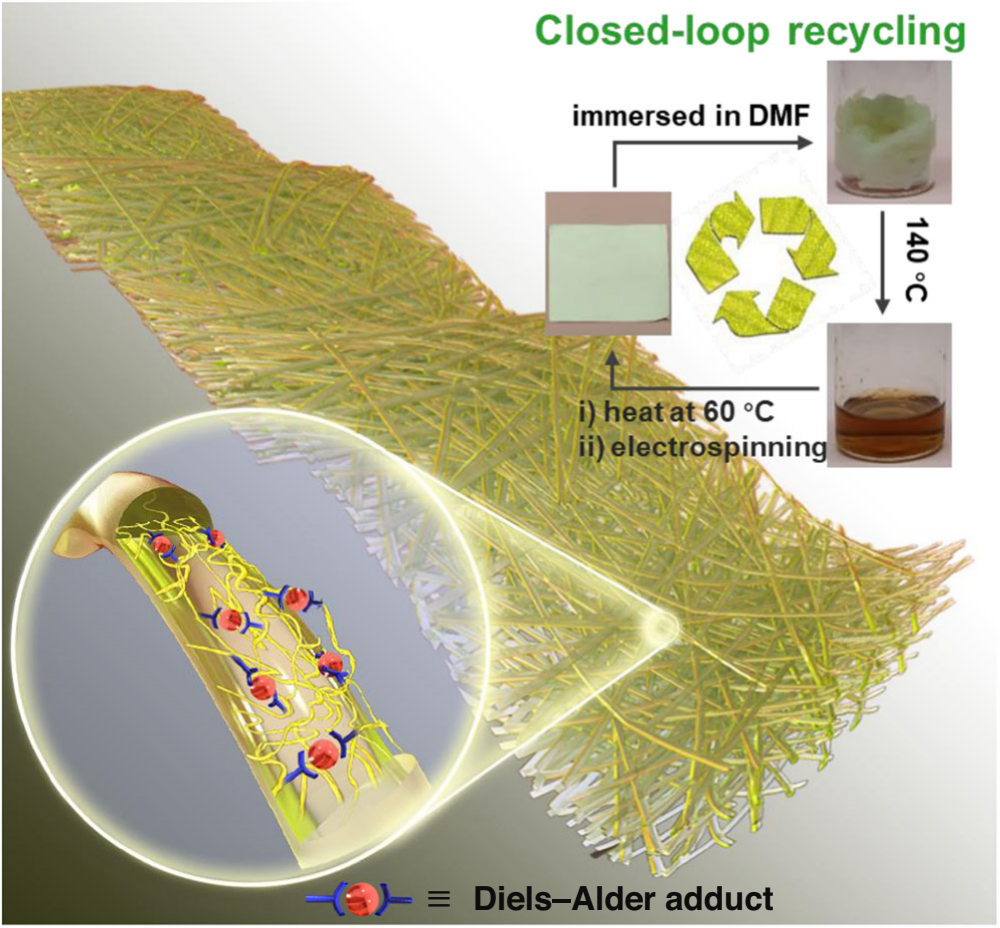
Overview of DCCNFs. Schematic representation of the DCCNFs with superior features and closed-loop lifecycle.

Herein, we demonstrate the concept of dynamic covalently crosslinked nanofibers (DCCNFs) by integrating the dynamic covalent crosslinking chemistry and nanofibers, aiming at high performance and closed-loop lifecycle of nanofibers. Figure 1 presents the overview of this work. DCCNFs were fabricated via a straightforward one-pot, in-situ crosslinking and catalyst-free electrospinning strategy by utilizing dynamic Diels–Alder (DA) cycloaddition reaction^36^ between furan and maleimide. The obtained DCCNFs exhibited homogeneous morphology, good flexibility, excellent mechanical properties, as well as dimensional, thermal and solvent stability. In addition, by means of the dynamic properties of the thermal response DA reaction in DCCNFs, the closed-loop recycling and welding of nanofibrous membranes could be readily achieved. We believe this strategy can be applied to other CANs to greatly enrich the family of nanofibers, and the developed DCCNFs can be further utilized in the fields of smart and sustainable materials.

## Results and discussion

### Fabrication and physical properties of DCCNFs

**Fig. 2:**
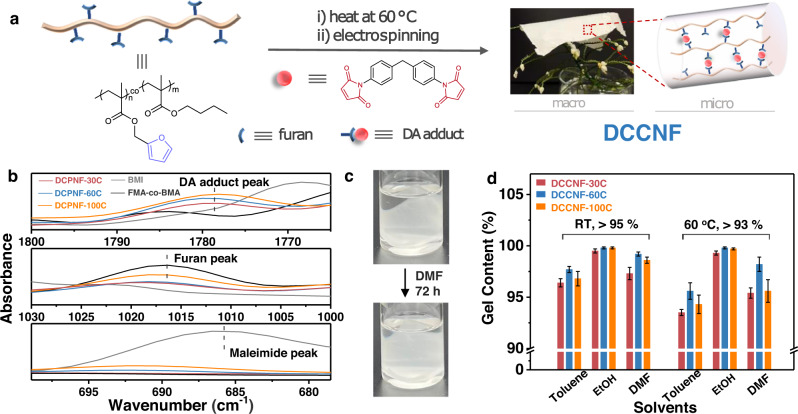
Fabrication of DCCNFs. **a** Fabrication route of DCCNFs and the schematic diagram of their crosslinking network; **b** FTIR spectra of FMA-co-BMA, BMI and DCCNFs in selected wavenumber range; **c** Digital photos of DCCNF-60C before and after soaking in DMF for 72 h; **d** Gel contents of DCCNFs in different solvents at RT and 60 °C.

To demonstrate the designed concept, a linear copolymer poly[(furfuryl methacrylate)-co-(butyl methacrylate)] (FMA-co-BMA, the molar ratio of FMA to BMA is 3:7) with pendant furan groups was prepared by free radical polymerization (Supplementary Fig. 1 and 2). BMA was chosen as a comonomer as the butyl group on the BMA side chain could provide flexibility and hydrophobicity to the target polymer. The M_n_ and M_w_ of FMA-co-BMA are around 40.1 K and 81.8 K, respectively (Supplementary Fig. 3). FMA-co-BMA in DMF or chloroform/methanol (v:v = 2:8) mixed solvents with different polymer contents were attempted for electrospinning. Pure FMA-co-BMA was not able to form uniform nanofibers with a large number of droplets and nodular nanostructure produced at either low or high concentrations (Supplementary Fig. 4)^37,38^. In contrast, in-situ crosslinking during electrospinning can endow polymer with the ability to form uniform nanofibers^13^. By simply mixing FMA-co-BMA with a bismaleimide (BMI, crosslinker) in DMF (25 wt.% of polymer in DMF), the viscosity of the mixed solution at room temperature can be easily regulated between 30-288 cps by heating at 60 °C within 55 minutes via Diels-Alder (DA) cycloaddition reaction (Supplementary Fig. 5). Then by electrospinning the mixed solutions with a viscosity of around 80 cps^39^, DCCNFs with different crosslinker loadings were successfully fabricated (Fig. 2a, Table 1 and Supplementary Table 1). During the electrospinning, solvents evaporate rapidly in high electric fields, and DA reaction between furan and maleimide occurs simultaneously, leading to in-situ crosslinking and formation of nanofibers^12,40^, as evidenced by the Fourier transform infrared spectroscopy (FTIR) spectra (Fig. 2b and Supplementary Fig. 6). Compared to FMA-co-BMA, the intensity of furan peaks around 1016 cm^−1^ (ring breathing) in DCCNFs decreases with the increasing BMI loading. The stretching vibration peaks of C = O in DA adduct around 1778 cm^−1^ appear, and their intensities also increase with the increase of BMI content, indicating the successful reaction between furan and BMI^41,42^. Besides, for sample DCCNF-30C, the characteristic peak of =C − H bending in maleimide around 685 cm^−1^ fully disappears, indicating the complete reaction between FMA-co-BMA and BMI. While for samples with high crosslinker loadings (DCCNF-60C and DCCNF-100C), the residues of the maleimide group are observed. This is due to the fact that the rigidity and steric hindrance of the polymer increases with the increase of BMI content, resulting in a decrease in the chance of active group contact. Nevertheless, this does not affect the formation of crosslinking networks. As shown in Fig. 2c, the obtained nanofibers could no longer be dissolved in DMF after 72 h soaking. Subsequently, three commonly used solvents (Toluene, ethanol, and DMF) were selected to calculate the gel contents of DCCNFs with different BMI loadings (Fig. 2d), which are all greater than 95% in the three solvents at RT. Furthermore, the gel contents of DCCNFs are all larger than 93%, even at 60 °C, indicating the formation of highly crosslinked networks^43^. Hypothetically, the gel content of DCCNF-100C with the largest crosslinker loading should be the highest, but it is lower than that of DCCNF-60C in the selected solvents and temperatures. This may be due to the increase in steric hindrance between the furan ring and the maleimide, as mentioned previously (Supplementary Fig. 6), resulting in a decrease in the crosslinking density.

**Table 1:** Physical and Mechanical Properties of different samples

Samples	Crosslinker Loadings (%)^[a]^	d_av_ (nm)^[b]^	S_q_^[c]^	Porosity (%)	WCA (°) ^[d]^	Tensile strength (MPa)	Young modulus (MPa)	Elongation at break (%)
DCCNF-30C	30%	404 ± 84	635	69.2 ± 2.3	118.6 ± 5.5	2.50 ± 0.24	52.5 ± 4.6	5.9 ± 0.8
DCCNF-60C	60%	339 ± 71	583	75.5 ± 4.1	129.0 ± 3.7	3.15 ± 0.15	79.2 ± 2.8	5.1 ± 0.3
DCCNF-100C	100%	419 ± 69	603	82.7 ± 2.5	129.4 ± 6.7	2.87 ± 0.08	118.5 ± 1.0	3.8 ± 0.2

[a] Molar ratio of maleimide to furan. [b] average fiber diameters. [c] root mean square roughness. [d] water contact angle.

**Fig. 3:**
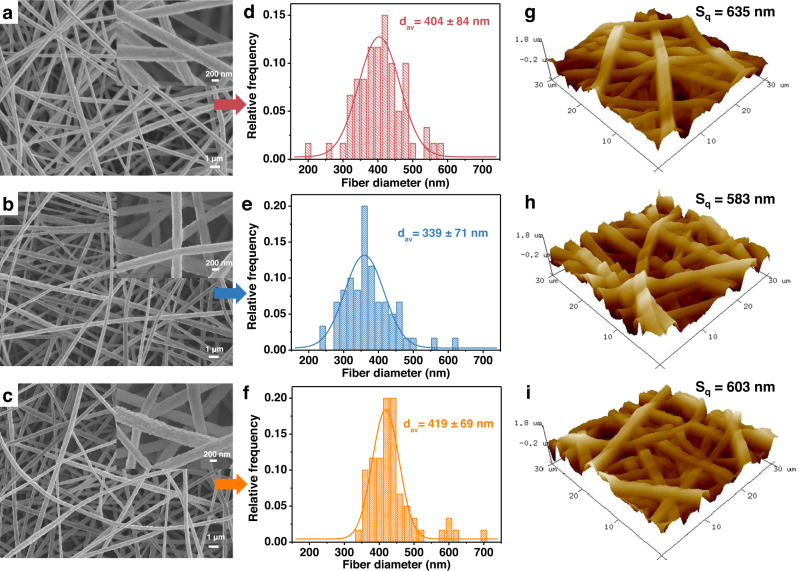
Morphology of DCCNFs. **a**–**c** FESEM images of A) DCCNF-30C, B) DCCNF-60C, and C) DCCNF-100C, with 5 K and 30 K magnifications; **d–f** The statistical distribution and average fiber diameters (d_av_) of D) DCCNF-30C, (**e**) DCCNF-60C, and (**f**) DCCNF-100C; **g–i** AFM images of (**G**) DCCNF-30C, (**h**) DCCNF-60C, and (**i**) DCCNF-100C, where S_q_ is root mean square roughness.

After determining the crosslinking chemistry of the polymers, a field emission scanning electron microscope (FESEM) was used to characterize the micromorphology of the nanofibers and illustrate the formation of nanostructures. As shown in Fig. 3a–c, uniformly distributed nanofibers with homogeneous morphologies are successfully obtained under different crosslinker loadings. By measuring and fitting the diameters of nanofibers in the SEM images, the statistical distribution and average fiber diameters (d_av_) of the DCCNFs can be obtained, which are between 339 and 419 nm (Fig. 3d–f and Table 1). To characterize the surface roughness of the prepared nanofibers, atomic force microscope (AFM) was used to measure the root mean square roughness (S_q_) of the nanofibrous membranes (Fig. 3g–i). The nanofibrous membranes fabricated with different crosslinker loadings possess similar surface roughness, with S_q_ ranging from 583 nm to 635 nm (Table 1). In addition, DCCNF membranes have high porosities, which are 69.2 ± 2.3%, 75.5 ± 4.1% and 82.7 ± 2.5% for DCCNF-30C, DCCNF-60C and DCCNF−100C, respectively (Table 1). Besides, benefiting from the hydrophobic butyl side chain, DCCNF membranes exhibit hydrophobic properties with the water contact angles (WCAs) of DCCNF-30C, DCCNF-60C and DCCNF−100C are 118.6 ± 5.5°, 129.0 ± 3.7°, and 129.4 ± 6.7°, respectively (Supplementary Fig. 7 and Table 1). Moreover, DCCNF membranes exhibit excellent flexibility and can be bent or worn on hand without breaking (Supplementary Fig. 8). These features enable the use of DCCNFs in the fields of the environment and electronics in the future^39,44^.

### Performance of DCCNFs

**Fig. 4:**
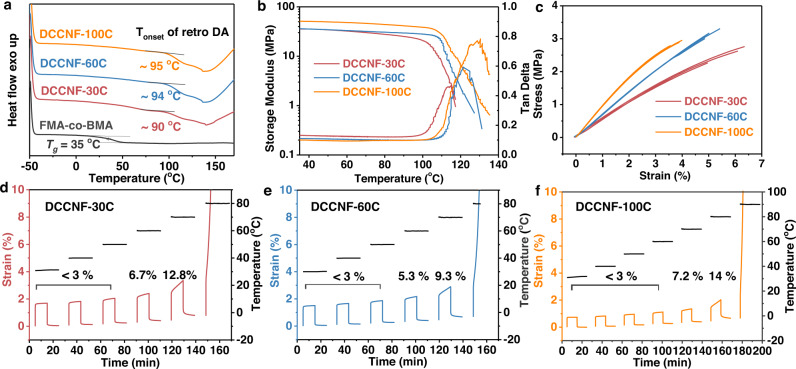
Thermo and mechanical performance of DCCNFs. **a** DSC thermograph of FMA-co-BMA and DCCNFs; **b** DMA curves of DCCNF membranes; **c** Stress-strain curves of DCCNF membranes; **d–f** Creep TTS curves and creep ratio data of (**d**) DCCNF-30C, (**e**) DCCNF-60C, and (**f**) DCCNF-100C.

In situ crosslinking not only endows polymers with processability but also offers them excellent properties. Thermal and mechanical properties of DCCNFs were characterized, which are also important parameters for nanofibers^45,46^. Figure 4a shows the differential scanning calorimetry (DSC) thermograph of FMA-co-BMA and DCCNFs, and the *T*_*g*_ of FMA-co-BMA is around 35 °C. However, after crosslinking, *T*_*g*_ cannot be observed in the DSC thermograph of DCCNFs, and only the retro DA reaction starting from around 90 to 95 °C for different samples is indicated^20^. In view of the undetected *T*_*g*_ of DCCNFs in DSC thermograph, a reasonable explanation is that the *T*_*g*_ of DCCNFs is higher than the temperature of retro DA. The retro DA reaction occurs earlier than *T*_g_, resulting in a change in the polymer structure and invisible *T*_*g*_ in DSC thermograph. This is further evidenced by the DMA curves, as the storage modulus of DCCNFs does not decrease significantly before 90 °C (Fig. 4b). These results indicate that the *T*_*g*_ of DCCNFs is much higher than that of the uncross-linked FMA-co-BMA due to the introduction of rigid structure and crosslinking points^16,47^. Besides, the crosslinking nature also enables DCCNF membranes to exhibit excellent mechanical properties with Young’s modulus ranging from 52.5 to 118.5 MPa, tensile strength ranging from 2.50 to 3.15 MPa, and elongation at break ranging from 3.8 to 5.9% (Fig. 4c and Table 1). The excellent mechanical strength is possibly due to i) the covalent crosslinking nature of nanofiber and ii) the complete of DA addition reaction within and on the surface of the nanofibers during the post-curing step. Besides, Young’s modulus increases and elongation at break decreases with the increasing crosslinker loading, while the tensile strength of DCCNP-100C is lower than that of DCCNP-60C, which does not conform to the laws of structural rigidity. This is probably ascribed to the crosslinking density and the increased internal stress produced by the post-curing process. Firstly, higher crosslink density endows DCCNF-60C with better tensile strength. Another factor is the internal stress, which is related to *T*_*g*_^48^: The internal stress will be low if the curing temperature is higher than *T*_*g*_; While the internal stress will be high if the curing temperature is lower than *T*_*g*_^43^. For DCCNP-100C and DCCNP-60C, their curing temperatures (60 °C) are both lower than their *T*_*g*_ (> 94 °C), and the higher rigid BMI loading leads to the lower reaction probability between furan and maleimide of DCCNP-100C. As a result, DCCNP-100C has high internal stress and comparatively low tensile strength. Moreover, we summarized the material properties of several electrospun nanofibrous membranes made from commercial polymers in literature and compared these with the developed DCCNF membranes. As show in Supplementary Table 2, the Young’s modulus of the DCCNF membrane is higher than those of existing nanofibrous membranes due to the presence of the crosslinked structures, and other properties of the DCCNF membrane are also comparable to them.

Due to the crosslinking nature, DCCNFs also exhibit superior dimensional, thermal, and solvent stabilities. Creep TTS experiments (Fig. 4d–f) were used to characterize the dimensional stability of DCCNF membranes. For DCCNF-30C and DCCNF-60C, their strain can be almost fully recovered below 50 °C (creep ratio <3%) after creep recovery experiments. In high temperatures (60 °C and 70 °C), DCCNF-30C and DCCNF-60C also present excellent creep resistance, with creep ratios of only 6.7% and 12.8% at 60 °C and 70 °C for DCCNF-30C, 5.3% and 9.3% at 60 °C and 70 °C for DCCNF-60C, respectively. Compared with DCCNF-30C, the dimensional stability of DCCNF-60C is more excellent, which is consistent with the law of crosslinking density^47,49^. DCCNF−100C with a more rigid structure presents better creep resistance with little creep below 60 °C, and only 7.2% and 14.2 % creep ratio at 70 °C and 80 °C, respectively. The thermal stability can be evaluated from the thermal stability of polymers and nanofibers. As shown in Supplementary Fig. 9, DCCNFs present a higher initial degradation temperature for 5% weight loss (*T*_*d5%*_) and char yield at 700 °C (*R*_*700*_) than uncross-linked FMA-co-BMA due to the introduction of aromatic rings in BMI. The DCCNF-100C with more aromatic rings loading presents higher *T*_*d5%*_ and *R*_*700*_ than other samples. For the thermal stability of nanofiber, DCCNF-60C was treated at different temperatures for 24 h, and their morphologies were imaged by FESEM (Supplementary Fig. 10). After 80 °C treatment, there is a marginal change in the nanofiber’s morphology compared to the original one. The nanofibers still maintain the original morphology well after 100 °C treatment, although slight adhesion of the contact parts of the nanofibers can be observed because the retro DA reaction is activated from around 94 °C (Fig. 4a). The adhesion area between nanofibers increases with the heating temperature, as evidenced by Supplementary Fig. 10. Nevertheless, the above results show that the DCCNFs can stably maintain the original morphology below 100 °C. Moreover, solvent, acid, and base stability were tested by immersing DCCNF-60C samples with dimensions 1*1 cm into different organic solvents, 1 M HCL and 1 M KOH, respectively. The macro (Supplementary Fig. 11) and micro (Supplementary Fig. 12) morphology before and after soaking were recorded. After 24 h soaking, the macroscopic appearances of DCCNF-60C in different solvents have neglectable change (Supplementary Fig. 11). FESEM was further used to characterize the microscopic morphology of the samples after solvent stability testing (Supplementary Fig. 12). In most organic solvents (DMF, MeOH, EtOH, and hexane), 1 M HCL, and 1 M KOH solutions, the micromorphology of the samples is well preserved. Besides, in acetone and toluene, adhesion between nanofibers was observed.

### Dynamic nature of DCCNFs

**Fig. 5:**
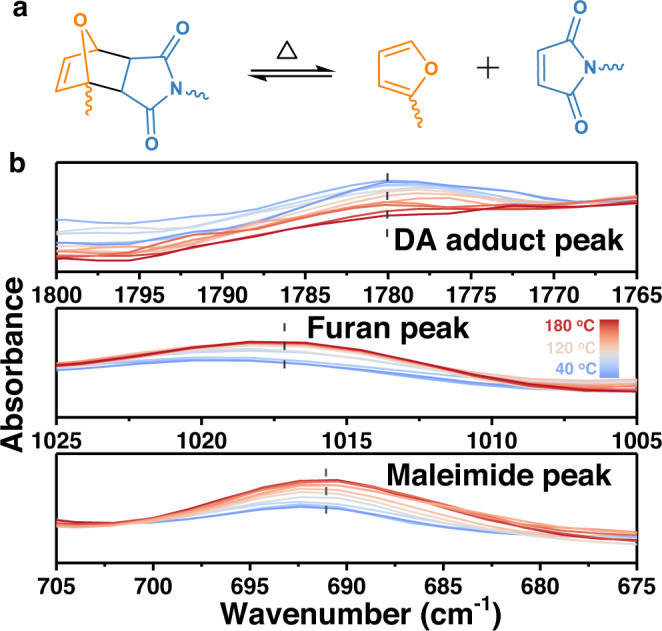
Dynamic nature of DCCNFs. **a** The reversible DA cycloaddition reaction. At low temperatures, furan reacts with maleimide to form DA adduct, while at high temperatures, the retro-DA reaction proceeds; **b** The real temperature FTIR spectra of DCCNF-60C from 40 °C to 180 °C.

The above results demonstrate the excellent performance of DCCNFs. However, not just nanofibers but all polymers, their life is limited due to inevitable performance degradation or macroscopic and microscopic fractures during usage, leading to a waste of resources and a heavy burden on the environment^19,50^. By employing the dynamic crosslinking property, DCCNFs may show unique functions, including closed-loop recycling and welding. The reversible DA cycloaddition reaction based on furan-maleimide structure is the most prominently explored and promising dissociative dynamic reaction and is widely used in the production of CANs^20,51^. At low temperatures, furan reacts with maleimide to form DA cycloadduct, while at high temperatures, the retro-DA reaction proceeds^52^ (Fig. 5a). In the present study, the successful combination of dynamic properties of DA adducts with DCCNFs was demonstrated by three different approaches, i.e, i) thermal transition in DSC thermograph, the endothermic peak of DA adducts depolymerization starts from around 90 to 95 °C (Fig. 4a). ii) bond characteristics in real-temp FTIR spectra (Fig. 5b), the characteristic peaks of furan ring breathing at 1016 cm^−1^, =C − H bending in maleimide at 685 cm^−1,^ and C = O stretching vibration in DA adduct at 1778 cm^−1^ do not change significantly under 90 °C; however, with the further increased temperature, the intensity of the characteristic peaks of =C − H bending in maleimide and furan ring breathing increase, and the peak intensity of C = O stretching vibration in DA adducts decrease. After reaching 150 °C, the intensity of the characteristic peaks no longer changes significantly (Fig. 5b). iii) molecular reaction evidenced by ^1^H NMR, 100 mg of DCCNF-60C was immersed in NMR tubes containing 0.7 ml of DMF-d7, and then heated at 140 °C. ^1^H NMR characterization was carried out after heating the tube for 3 min, 5 min, 10 min and 20 min, respectively. As shown in the ^1^H NMR spectra (Supplementary Fig. 13a), after heating for 3 minutes, samples partially dissolved in DMF-d7, and the characteristic peaks of the proton in the double bonds of furan and maleimide after retro-DA reaction are observed. When heated to 5 min, the sample has been completely dissolved, and the ^1^H NMR spectrum no longer produces significant changes with further heating. Supplementary Fig. 13b shows the detailed ^1^H NMR spectrum of DCCNF-60C after heating at 140 °C for 5 min. Obviously, the chemical shift corresponds to the structure of the polymer and BMI. Besides, by comparing the characteristic peaks of A1 and B1, B2, the ratio of the furan ring to maleimide is 1:0.54, which is close to the theoretical maleimide loading (1:0.6), indicating that DCCNF can be completely degraded into the original mixture in DMF. The above three pieces of evidence strongly prove the thermal reversibility of the developed nanofibers.

### Closed-loop recycling and welding of DCCNFs

**Fig. 6:**
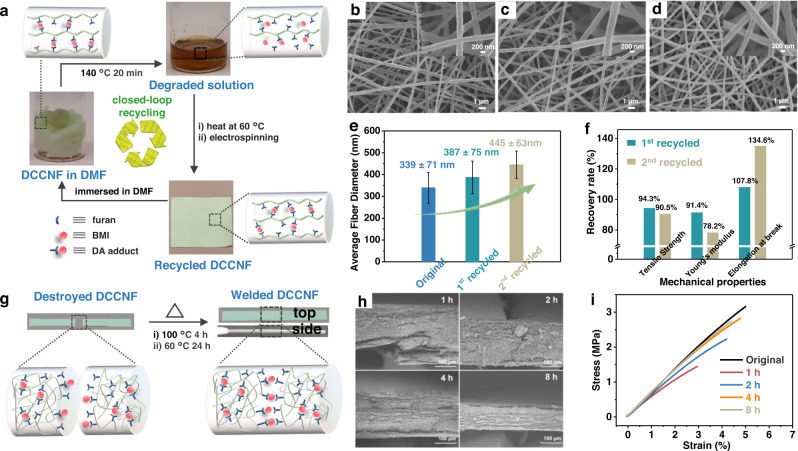
Closed-loop recycling and welding of DCCNFs. **a** Schematic representation of the one-pot closed-loop recycling process of DCCNF-60C; **b**–**d** FESEM images of **b**) original, **c**) 1^st^ recycled, and **d**) 2^nd^ recycled DCCNF-60C; **e** Average fiber diameters of original and recycled DCCNF-60C; **f** Recovery rate of mechanical properties after each recycling cycle of DCCNF-60C; **g** Schematic representation of the welding process of DCCNF-60C; **h** FESEM images of welded DCCNF-60C with different heating durations at 100 °C; **I** Stress-strain curves of the original and welded DCCNF-60C with different heating durations at 100 °C.

Thanks to the dynamic crosslinking nature, DCCNFs may present excellent one-pot closed-loop recyclability. 0.8 g of DCCNP-60C was placed in a sample vial containing 2.4 ml DMF (25 wt% of polymer concentration, same as in DCCNF-60C formation step), and after heating at 140 °C for 20 min, DCCNF-60C was fully degraded in DMF due to the retro-DA reaction (Fig. 6a). After heating the degraded solution until the viscosity reached around 80 cps, recycled nanofibers could be fabricated by in-situ crosslinking electrospinning again (Fig. 6a). In theory, such a simple one-pot closed-loop recycling strategy can give DCCNF an infinite lifecycle. Here we demonstrate its excellent closed-loop recycling capability by recycling DCCNF-60C for two cycles. FTIR (Supplementary Fig. 14) shows that the recycled nanofibers retain the original chemical structure, as there is no noticeable change in the characteristic peaks of =C − H bending in maleimide at 685 cm^−1^, furan ring breathing at 1016 cm^−1,^ and C = O stretching vibration in DA adduct at 1778 cm^−1^. In addition, the recycled DCCNF-60C also preserves the original morphology with homogeneously distributed nanofibers (Fig. 6b-d). There is only a slight increase in average fiber diameter with the increased number of recycling times (Fig. 6e and Supplementary Fig. 15). In terms of mechanical properties (Fig. 6f, Supplementary Fig. 16 and Supplementary Table 2), the recycled DCCNF-60C also performs very well, with the recovery rates of tensile strength and Young’s modulus for the 1^st^ recycle cycle reaching 94.3% and 90.5%, respectively. After the second recycling cycle, the recovery rate of tensile strength still achieves more than 90%, but the decrease in Young’s modulus is observed with a 78.2% recovery rate. The decrease in stiffness is coupled with an increase in toughness, with the recovery rate of elongation at break reaching 107.8% and 134.6% after the first and second recycling cycle, respectively. The main parameters that influence the mechanical characteristics of nanofibrous membranes include fiber orientation, diameter and diameter distribution, and polymer structure etc.^53,54^ The original and recycled nanofibers are all anisotropic, and FTIR spectra (Supplementary Fig. 14) show that the chemical structure of the polymer does not change significantly, suggesting the mechanical properties of DCCNF membrane are primarily impacted by the diameters of the nanofibers. After recycling, the diameter of the nanofibers increases (Fig. 6e), resulting in a decrease in mechanical strength and modulus, and an increase in elongation at break. Besides, 10 cycles closed-loop recycling of DCCNF-60C were performed. The stress-strain curves of the recycled DCCNF-60C during the third to tenth cycles are shown in Supplementary Fig. 17, and the results are presented in Supplementary Table 3. Overall, one can see that the modulus of the nanofibrous membrane showed a trend of decreasing first and then increasing. Correspondingly, the elongation at break first increased and then decreased. In addition, the tensile strength showed a decreasing trend. One explanation for the observed trend is that in the few recycling cycles, the diameter of the nanofibers increased, resulting in a decrease in the modulus, and in subsequent recycling cycles, due to repeated high-temperature treatments, the polymer inevitably undergoes some side reactions (e.g. partial self-polymerization and oxidation of furan and maleimide double bonds), resulting in permanent crosslinking, and thus leading to an increase in Young’s modulus. Moreover, the thermal properties (Supplementary Fig. 18) and thermomechanical behavior (Supplementary Fig. 19) of DCCNF-60C do not change significantly after each cycle. These results demonstrate the excellent closed-loop recycling capability of DCCNFs.

Subsequently, we explored the weldability of DCCNF, which could also be achieved by the dissociation and association of dynamic DA reaction (Fig. 6g). In the DSC thermograph (Fig. 4a), the one-set temperature of DA adduct dissociation in DCCNF-60C is about 94 °C, and in the thermal stability experiment, the adhesion of nanofibers is observed at 100 °C (Supplementary Fig. 10). To avoid side reactions at high temperatures, we chose 100 °C as the experiment temperature to verify the weldability of DCCNF-60C. Two fragments of DCCNF-60C were laterally stacked together and held in an oven at 100 °C with two glass sheets pressing against the membrane for a range of heating durations. After that, the samples were heated at 60 °C for 24 h to ensure the association of partially dissociated DA adducts (Fig. 6g). After welding, the broken fragments of DCCNF-60C are firmly bonded without fracture surface from both the top and side views (Fig. 6g). Figure 6h presents the FESEM images of the top view of welded DCCNF-60C with different heating durations at 100 °C. It can be clearly seen that the cracks between the pieces shrink over time, and the two pieces are fully bonded in 4 h. Moreover, tensile behaviors of DCCNF-60C before and after welding were studied (Fig. 6h), and the recovery rates after different treating times at 100 °C were recorded in Supplementary Fig. 20. After heating at 100 °C for 1 h, DCCNF can recover 62.5%, 46%, and 58.2% of Young’s modulus, tensile strength, and elongation at break, respectively. After 2 h-heating, the recovery rates increase to 83.3%, 71.1%, and 82.7% for Young’s modulus, tensile strength, and elongation at break, respectively. After 4 h heating, DCCNF-60C can almost restore the original mechanical properties with more than 90% recovery rates for all three parameters of mechanical properties. Besides, the mechanical properties of DCCNF-60C no longer change significantly with extended heating time. These results elucidate the excellent closed-loop recycling and welding abilities of DCCNFs.

In summary, we propose the concept of dynamic covalently crosslinked nanofibers (DCCNFs) through the successful unification of covalent adaptable networks chemistry and nanofiber technology, which yields an excellent combination of high performance and sustainability for nanofibers. The DCCNFs can be readily fabricated by viscosity modulation and in-situ crosslinking based on the catalyst-free DA reaction between a furan-suspended linear copolymer and bismaleimide via the electrospinning process. The produced DCCNF membranes present homogeneous macro and micro morphologies, high porosity, hydrophobicity, and good flexibility. Besides, FTIR spectra and gel content prove the successful formation of DA adduct and highly crosslinked networks, which endows DCCNF membranes with excellent thermal and mechanical properties as well as dimensional, thermal, and solvent stabilities. Moreover, the wear-and-tear problems, such as performance degradation, macroscopic and microscopic fractures, can be solved due to the dynamic dissociation and association of the thermal-reversible Diels-Alder reaction. DCCNFs exhibit excellent one-pot closed-loop recycling and post-fracture welding capabilities with negligible change in morphology and performance after recycling and welding. The robust and flexible DCCNFs with one-pot closed-loop recyclability and weldability may have great potential for electronics, energy and environmental applications.

## Methods

### Synthesis of Poly[(furfuryl methacrylate)-co-(butyl methacrylate)] (FMA-co-BMA)

FMA-co-BMA was synthesized by free radical polymerization^55^. FMA (4 g, 0.024 mol), BMA (7.96 g, 0.056 mol), and free radical initiator AIBN (0.13 g, 0.0008 mol) were dissolved in 50 mL toluene using a 250 mL flask. The solution was stirred and purged with argon for 40 minutes at room temperature to ensure that all air was expelled. Subsequently, the solution was heated to 70 °C and reacted for 24 h. After the reaction, FMA-co-BMA was obtained by precipitation in methanol for four times and then dried in a vacuum oven.

### Fabrication of dynamic covalently crosslinked nanofibers (DCCNFs)

DCCNFs were prepared by electrospinning^1^. 0.8 g FMA-co-BMA was dissolved in 2.4 g DMF (25 wt.% of polymer) in a 20 ml sample bottle. Then BMI with a different molar ratio to furan in FMA-co-BMA (Supplementary Table 1) was added into the solution and dissolved by vigorous shaking. Additionally, DMF was added to the solution to ensure that the total concentration of the solution was 25% (w/w). Then the solution was heated at 60 °C until the viscosity reached around 80 cps at room temperature. Subsequently, the solution was sucked into a 5 mL syringe with a blunt 22-gauge needle. After evacuating the air from the syringe, the solution was electrospun at a constant rate of 1 mL h^−1^ with a voltage of 16 kV. The nanofibers were collected on an aluminum foil (collector) with a gap of 20 cm, followed by drying and post-curing at 60 °C for 24 h before use.

### Gel Content Test

Different samples (around 200 mg) were separately placed in various solvents at RT or 60 °C for 72 h and then washed with the same solvents and dried in a vacuum oven for 24 h. The gel content (%) is calculated by the following formula: 100 × m_1_/m_0_, where m_0_ is the original mass before the test, and m_1_ is the final mass after drying.

### Closed-loop Recycling Process

0.8 g DCCNF-60C was put into 2.4 ml DMF (25 wt.% of polymer content), followed by heating at 140 °C for 20 min. DCCNF-60C was fully degraded in DMF by retro-DA reaction. After the solution was rapidly cooled to room temperature in water, it was heated at 60 °C until the viscosity reached around 80 cps at room temperature. Then the solution was sucked into a 5 mL syringe with a blunt 22-gauge needle and fabricated using electrospinning as in the original process to obtain the recycled DCCNF-60C. For 4^h^-7^th^ and 8^th^-10^th^ recycling cycles, polymer concentration was adjusted to 20% and 15%, respectively, and other parameters were the same for each recycling cycle.

### Weldability Experiments

Two fragments of the cut DCCNF-60C were placed closely and sandwiched by two glasses to ensure good contact. Then, the samples were heated at 100 °C in an oven for different times to study morphology and mechanical properties under different bonding times. After that, the welded samples were heated at 60 °C for 24 h to ensure the association of partially dissociated DA adduct.

## Supplementary information

Supplementary Information

## Data Availability

The authors declare that all data supporting this work are available within the paper and its Supplementary Information files.
