# Supplementary material for: In-situ forming dynamic covalently crosslinked nanofibers with one-pot closed-loop recyclability
Source: Nat Commun. 2023 Mar 2;14:1182. doi: 10.1038/s41467-023-36709-4 (PMC9981754; doi:10.1038/s41467-023-36709-4)
Supplement: Supplementary file 1 — Supplementary Information [file 41467_2023_36709_MOESM1_ESM.pdf]

In-situ forming dynamic covalently crosslinked nanofibers with one-pot closed-loop recyclability

Sheng Wang[1], Nannan Wang[1], Dan Kai[1,2], Bofan Li[1]*, Jing Wu[2], Jayven Chee Chuan YEO[2], Xiwei Xu[3], Jin Zhu[3], Xian Jun Loh[2], Nikos Hadjichristidis[4]*, Zibiao Li[1,2,5]*

[1]Institute of Sustainability for Chemicals, Energy and Environment (ISCE[2]), Agency for Science, Technology, and Research (A*STAR), Singapore 627833, Singapore. [2]Institute of Materials Research and Engineering, Agency for Science, Technology, and Research (A*STAR), Singapore 138634, Singapore. [3]Key Laboratory of Bio-Based Polymeric Materials Technology and Application of Zhejiang Province, Ningbo Institute of Materials Technology and Engineering, Chinese Academy of Sciences (CAS), Ningbo 315201, P. R. China. [4]Polymer Synthesis Laboratory, Physical Sciences and Engineering Division, KAUST Catalysis Center, King Abdullah University of Science and Technology (KAUST), Thuwal, 23955, Saudi Arabia. [5]Department of Materials Science and Engineering, National University of Singapore, Singapore 117576, Singapore. *Corresponding author, email: li_bofan@isce2.a-star.edu.sg (B. Li); Nikolaos.Hadjichristidis@kaust.edu.sa (N. Hadjichristidis); lizb@imre.a-star.edu.sg (Z. Li)

This file includes:

1. Supplementary Methods P2-3
2. Supplementary Figures P4-13
3. Supplementary Tables P14-15
4. Supplementary References P15

1. Supplementary Methods

Materials. Furfuryl methacrylate (FMA, 97%), 1,1′-(Methylenedi-4,1-phenylene)bismaleimide (BMI, 95%), 2,2′-Azobis(2-methylpropionitrile) (AIBN, 98%), toluene (anhydrous, 99.8%), N,N-Dimethylformamide (anhydrous, 99.8%), methanol (ACS reagent, ≥99.8%) were purchased from Sigma-Aldrich. Butyl methacrylate (BMA, >99%) were purchased from Tokyo Chemical Industry. All chemicals were used as received.

Characterizations. Nuclear magnetic resonance (NMR) spectra were performed on a JEOL JNM-ECA500II FT NMR System by using $CDCl_3$ or DMF-d7 as the solvent. Agilent 1260 Infinity II GPC/SEC System with refractive index detector was used to perform size exclusion chromatography (SEC). Calibration was done using monodisperse polystyrene (PS) standards. HPLC-grade THF was used as the eluent at a flow rate of 1.0 mL min^{-1}. The viscosity of the solution for electrospinning were performaced on a Anton Paar VisoQC100 rotational viscometer with a 100 rpm rotational speed. The Fourier transform infrared spectra (FTIR) were measured with the High-Resolution FTIR (Bruker Vertex 80v) by attenuated total reflection (ATR) mode. For real-temperature FTIR, a temperature control accessory was used. The micromorphology of different samples was imaged by a JEOL JSM 6700F field emission scanning electron microscope (FESEM). Before imaging, the samples were coated with a thin layer of gold using a gold sputtering equipment (Jeol JFC-1200). The fiber diameters were calculated from 60 random measurements per FESEM image by the ImageJ program. The surface roughness of the fiber samples was evaluated using Atomic Force Microscope (Bruker Dimension ICON) in the standard tapping mode (in the air), with a scanning rate at 0.5 Hz and spot size of 30 x 30 µm. The roughness value, S_q, were derived from the average of five S_q values. The porosity of the nanofibers was measured by filling the pores of a pre-weighed substrate sample using isopropanol with a density 0.786 g mL^{-1}. The samples were weighed before and after IPA immersion, and the porosity was calculated using the equation

$$Porosity = \frac{W_w - W_d}{Ad\rho}$$

where A is the surface area of the sample, d is the average thickness of the membrane, ρ is the IPA density, W_w and W_d are the mass of the wet and dry samples, respectively. Differential scanning calorimetry (DSC) was implemented using a Mettler Toledo DSC1 under an N_2 atmosphere. Samples around 10 mg were heated from -50 to 150 °C at a heating rate of 10 °C min^{-1} and kept at 150 °C for 2 min, followed by being cooled to

-50 °C at a cooling rate of 10 °C min^{-1}, and then heated to 170 °C at a heating rate of 10 °C min^{-1}. A TA Q800 Dynamic Thermomechanical Analysis (DMA) machine was used for the temperature sweep test, tensile creep TTS test and stress-strain test with sample dimensions of around 20 mm (length) × 5 mm (width) × 0.1 mm (thickness). For the temperature sweep test, from 30 to 120 °C with a heating rate of 5 °C min^{-1} at a frequency of 1Hz, the storage modulus, loss modulus and tan delta data were recorded. For the tensile creep TTS test, experiments were conducted at temperatures from 30 °C to an interval of 10 °C, and a constant force of 1 MPa was applied for 10 min followed by a 10 min recovery period during the measurement of each isotherm. The stress-strain tests were tested with an elevated force of 3 N min^{-1}. The tensile properties of each sample were reported as the average of three measurements. Thermogravimetric analysis (TGA) was conducted on a TA Instruments TGA Q500 from 50−700 °C at a heating rate of 10 °C min^{-1} under a N_2 atmosphere with approximately 5 mg of different samples.

2. Supplementary Figures

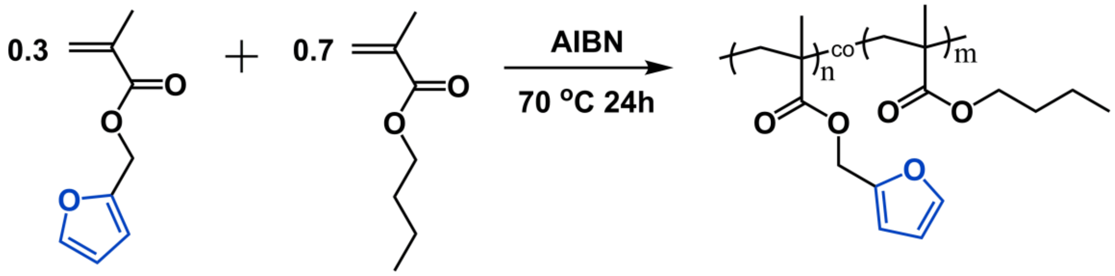

Supplementary Fig.1 Synthetic route of poly[(furfuryl methacrylate)-co-(butyl methacrylate)] (FMA-co-BMA).

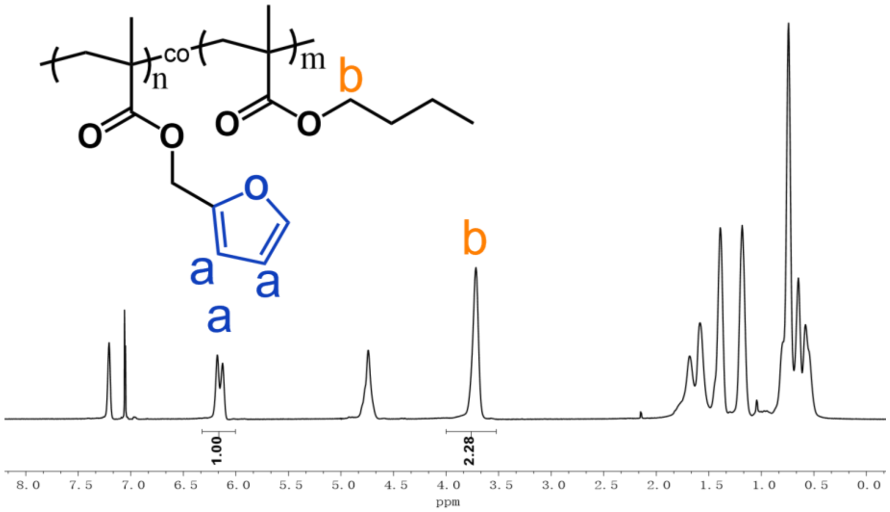

Supplementary Fig.2 ^1H NMR ($CDCl_3$, 500 MHz, 25 °C) spectrum of FMA-co-BMA.

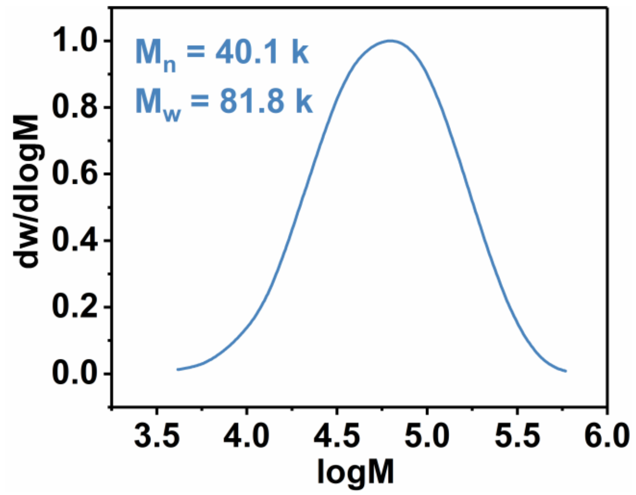

Supplementary Fig.3. SEC trace of FMA-co-BMA (THF, 40 °C, PS standards).

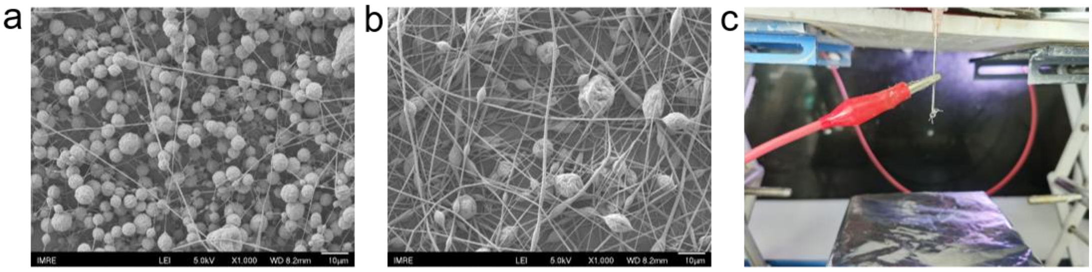

Supplementary Fig.4. (a, b) SEM images obtained by electrospinning of FMA-co-BMA with different concentrations: 35 wt% in DMF (a); 30 wt% in chloroform/methanol (b); (c) Typical photo of electrospinning of FMA-co-BMA at higher concentration in DMF or chloroform/methanol mix solution.

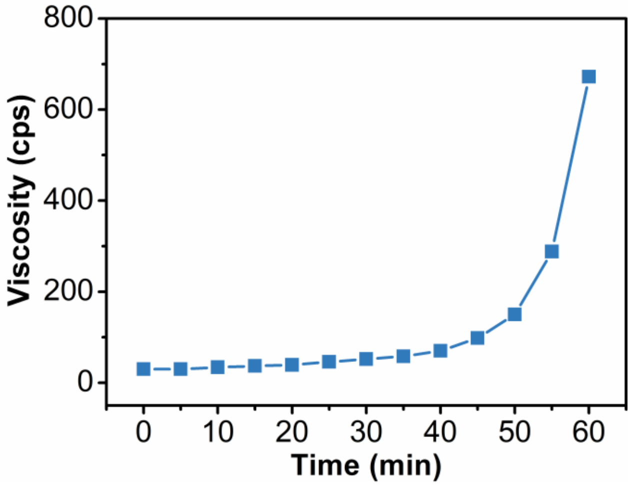

Supplementary Fig.5 The viscosity of the mixed solution (25 wt.% of DMF FMA-co-BMA and bismaleimide in DMF, the molar ratio of maleimide to furan is 0.6:1) at room temperature by heating at 60 °C for different time.

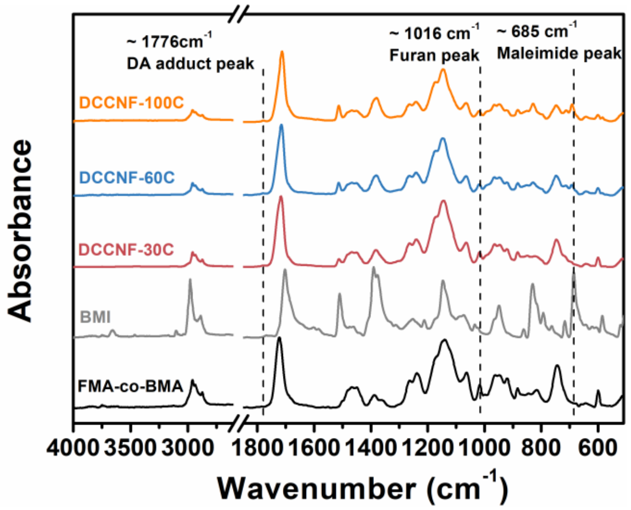

Supplementary Fig.6. FTIR spectra of linear polymer FMA-co-BMA, crosslinker BMI and DCCNFs.

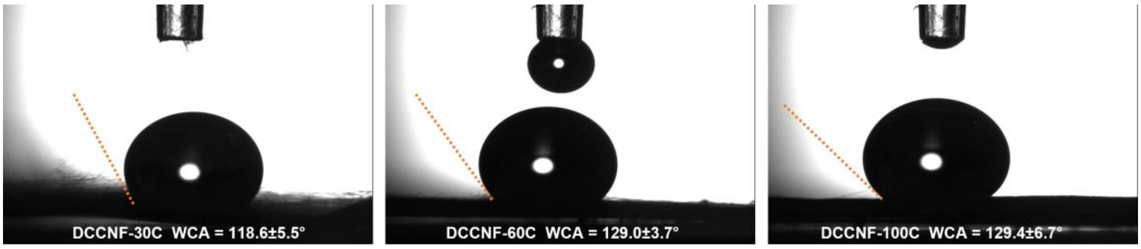

Supplementary Fig.7. Water contact angle (WCA) images of different samples.

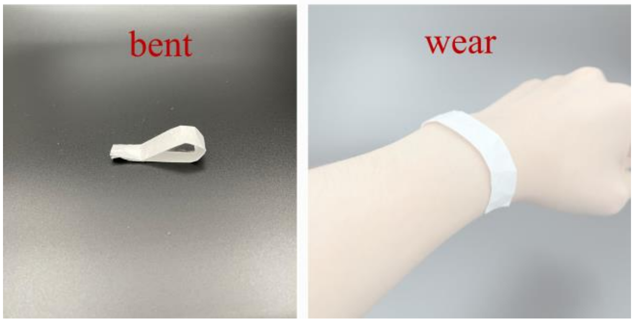

Supplementary Fig.8. Digital photos of bent DCCNF membranes or worn by hand.

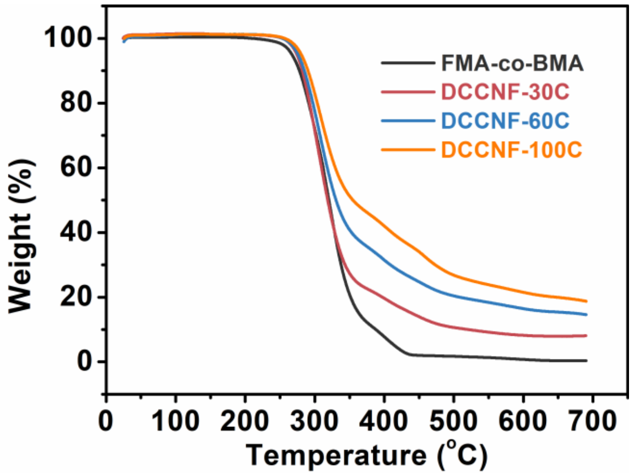

Supplementary Fig.9. TGA curves of different samples.

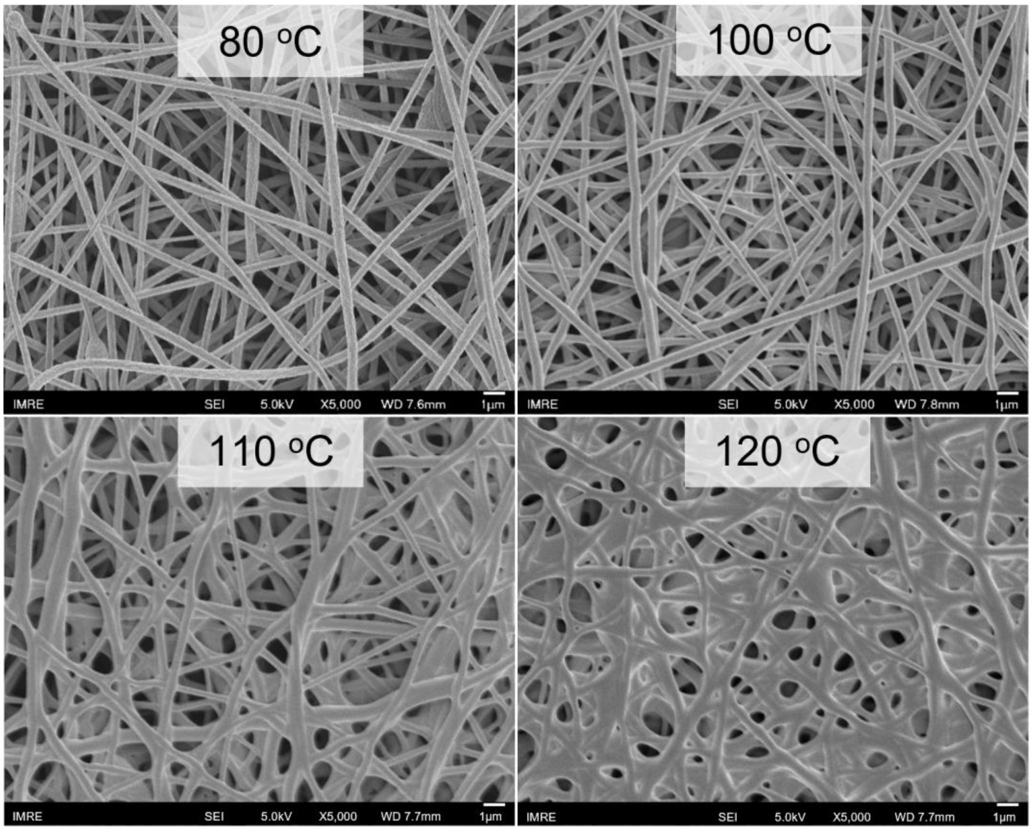

Supplementary Fig.10. SEM image of DCCNF-60C after heat treatment at different temperatures.

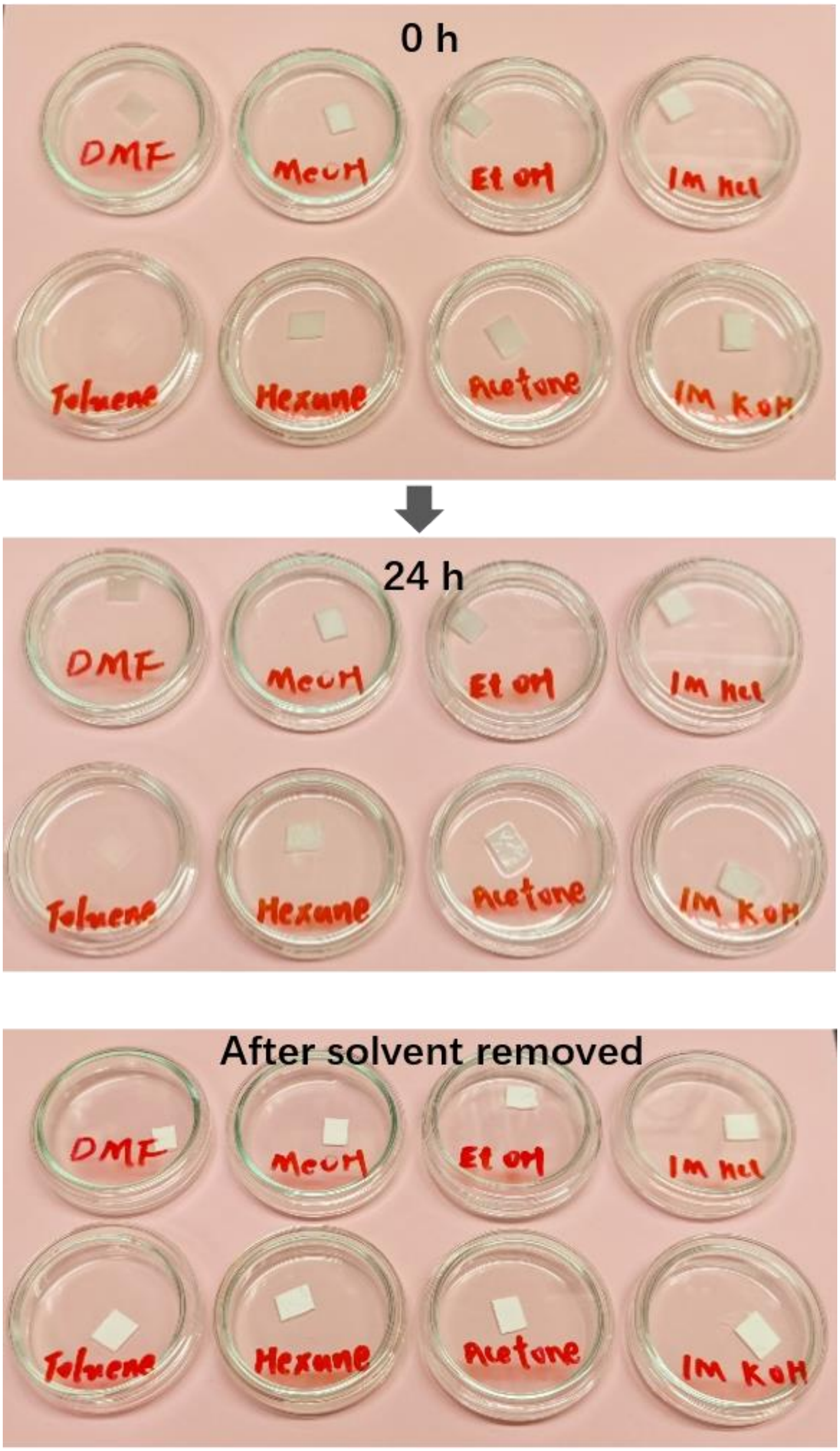

Supplementary Fig.11. Digital photos of DCCNF-60C before and after solvent soaking.

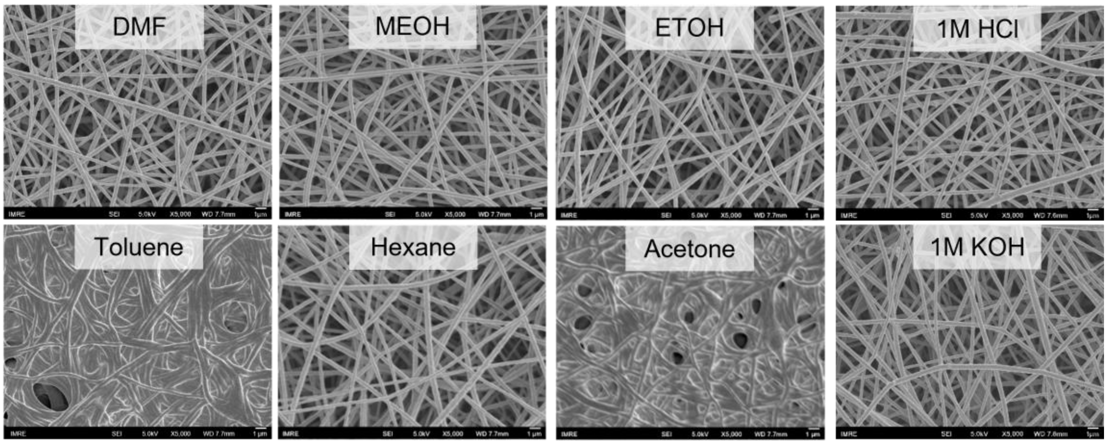

Supplementary Fig.12. SEM images of DCCNF-60C after solvent soaking.

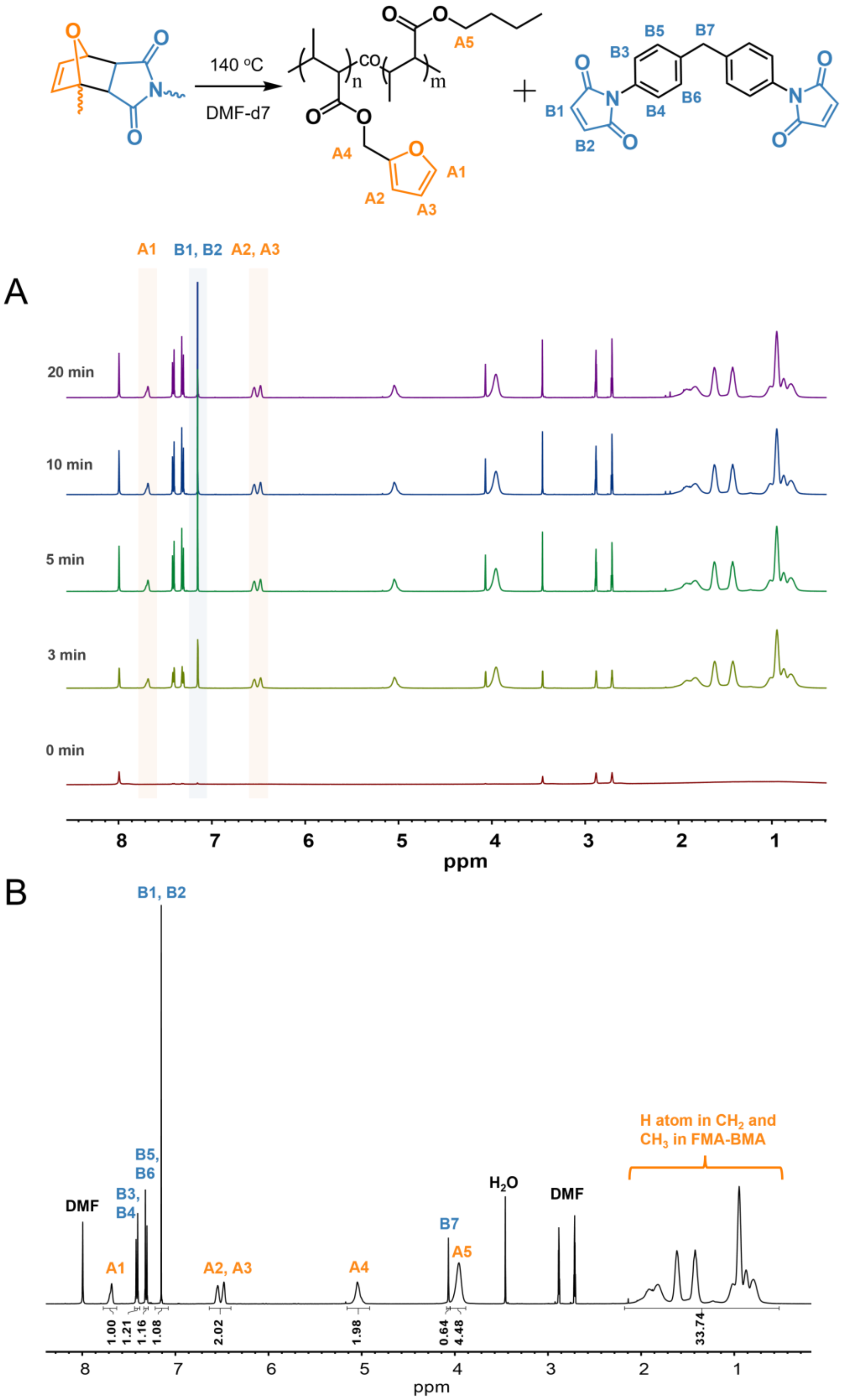

Supplementary Fig.13 ^{1}H NMR (DMF-d7, 500 MHz, 25 °C) spectra of 100 mg DCCNF-60C in 0.7 ml of DMF-d7 after heating at 140 °C for A) different durations, and B) 5 min.

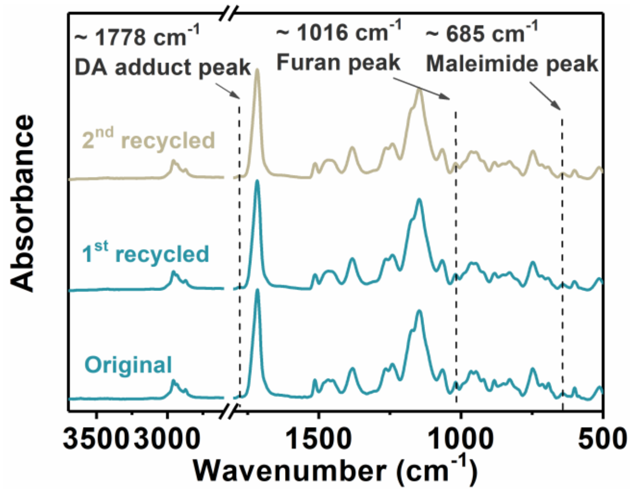

Supplementary Fig.14. FTIR spectra of original and two cycles recycled DCCNF-60C.

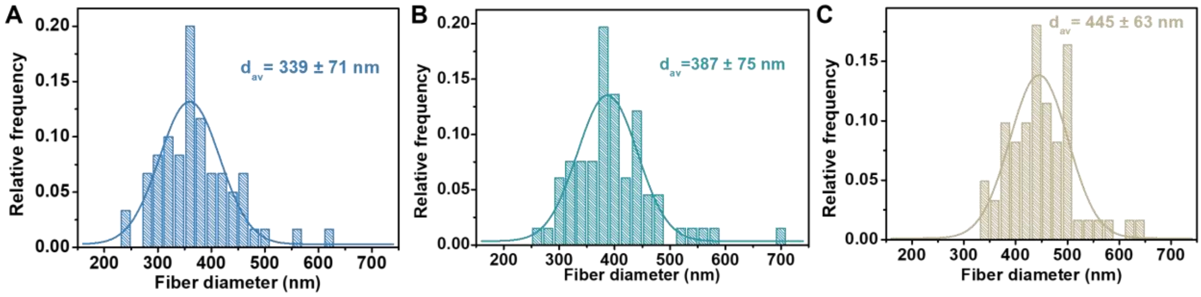

Supplementary Fig.15. The statistical distribution and average fiber diameters of A) original, B) 1st recycled and C) 2nd recycled DCCNF-60C.

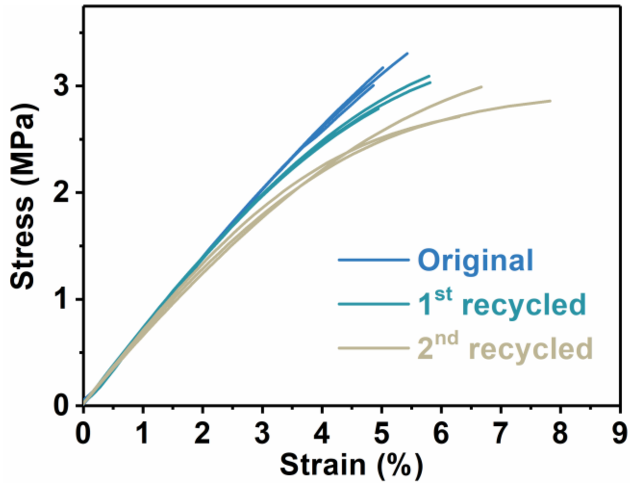

Supplementary Fig.16. Stress-strain curves of original and two cycles recycled DCCNF-60C.

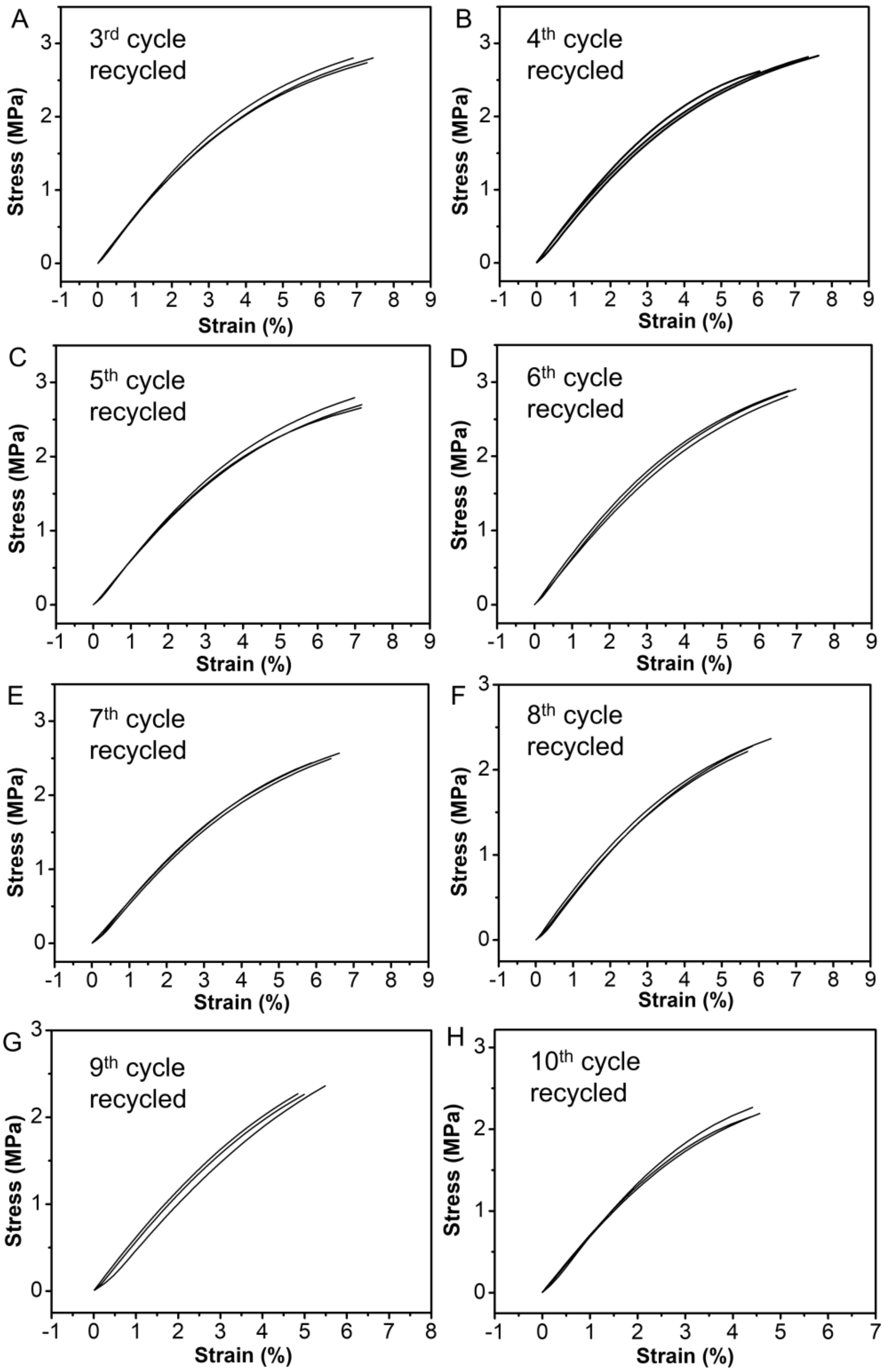

Supplementary Fig.17 Stress-strain curves of the A) 3rd, B) 4th, C) 5th, D) 6th, E) 7th, F) 8th, G) 9th and H) 10th cycles recycled DCCNF-60C.

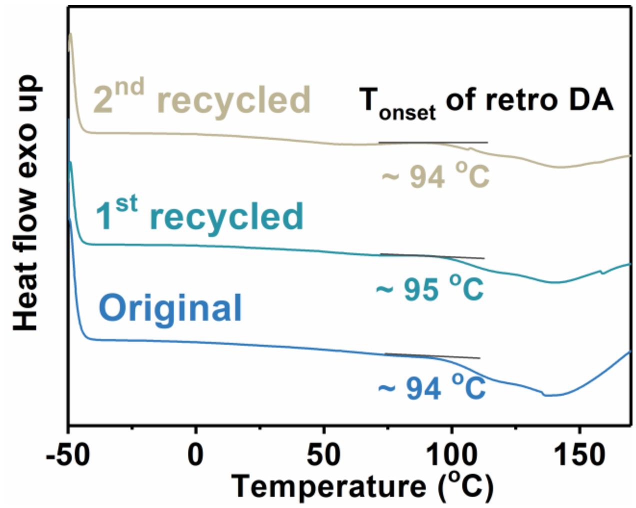

Supplementary Fig.18. DSC thermograph of original and two cycles recycled DCCNF-60C.

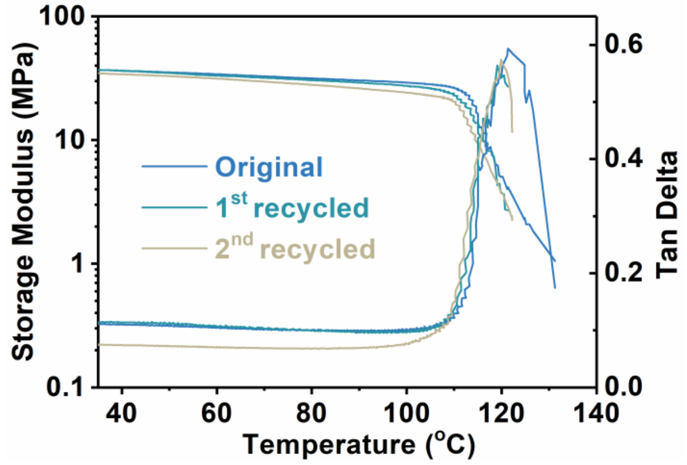

Supplementary Fig.19. DMA curves of original and two cycles recycled DCCNF-60C.

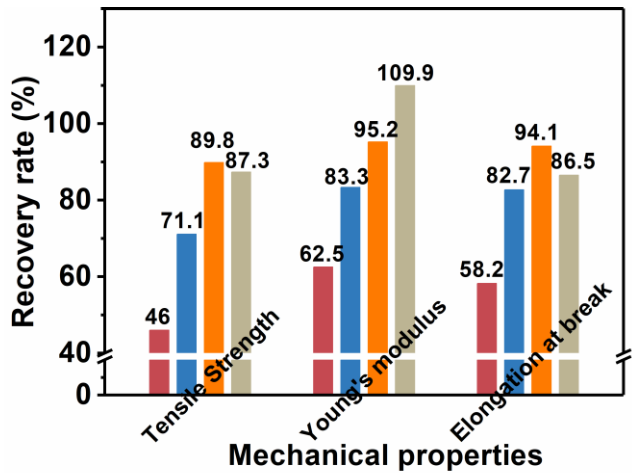

Supplementary Fig.20. Recovery rate of mechanical properties after welding at 100 °C for 1 h, 2 h, 4 h and 8 h.

3 Supplementary Tables

Supplementary Table 1 Electrospinning feed ratio of different samples

Sample name	FMA-co-BMA (g)	BMI (g)	Molar ratio of maleimide to furan	solvent	Polymer concentration (wt%)
DCCNF-30C	0.8	0.088	30%	DMF	25%
DCCNF-60C	0.8	0.175	60%	DMF	25%
DCCNF-100C	0.8	0.292	100%	DMF	25%

Supplementary Table 2 Material properties of DCCNFs compared to commercial polymer-based nanofibrous membranes

Nanofibrous membranes	d_{av} (nm)[b]	Porosity (%)	WCA (°) [d]	Tensile strength (MPa)	Young modulus (MPa)	Elongation at break (%)	Ref.
DCCNF-30C	404	69.2±2.3	118.6±5.5	2.50±0.24	52.5±4.6	5.9±0.8	This work
DCCNF-60C	339	75.5±4.1	129.0±3.7	3.15±0.15	79.2±2.8	5.1±0.3	This work
DCCNF-100C	419	82.7±2.5	129.4±6.7	2.87±0.08	118.5±1.0	3.8±0.2	This work
PAN	210	65.9±1	75	11.5	-	16.8	1
PAN/CA	303-502		126	1.4-5.1	0.5-1.3	14.8-29.2	2
PVDF	252	-	-	5.53	14.54	64	3
PVDF-co-HFP	360	85.4	136.7	4.1	3.33	84	4
PVP	305	-	-	2.3	-	9.1	5
PLA	609	71.8	139.6	42.34	1.27	-	6
PVA	300	84	-	4.8	6.4	174	7
PVA	240	-	-	15.6	58.2	85.5	8
TPU	-	-	110	7.08	2.03	408	9
ABS	530	-	134.8	1.1	14.6	55	10

[a] Molar ratio of maleimide to furan. [b] average fiber diameters. [c] root mean square roughness. [d] water contact angle. PAN: polyacrylonitrile. CA: cellulose acetate. PVDF: poly(vinylidenefluoride). HFP: hexafluoropropylene. PVP: poly (vinyl pyrrolidone). PLA: polylacticacid. PVA: polyvinyl alcohol. TPU: Thermoplastic polyurethane. ABS: Acrylonitrile butadiene styrene.

Supplementary Table 3 Mechanical properties data and recovery rate of original and recycled DCCNF-60C

Samples	Tensile strength (σ, MPa)	Recovery rate of σ	Young modulus (E, MPa)	Recovery rate of E	Elongation at break (ε, %)	Recovery rate of ε
Original	3.15±0.15	-	79.2±2.8	-	5.1±0.3	-
1st recycled	2.97±0.21	94.3%	72.2±2.5	91.2%	5.5±0.6	107.8%
2nd recycled	2.85±0.14	90.5%	61.8±4.4	78.0%	6.9±0.9	135.3%
3rd recycled	2.78±0.04	88.3%	61.1±3.1	77.1%	7.2±0.3	141.2%
4th recycled	2.75±0.12	87.3%	59.8±4.2	75.5%	7.0±0.9	137.3%
5th recycled	2.72±0.07	86.3%	57.8±3.6	73.0%	7.1±0.1	139.2%
6th recycled	2.87±0.05	91.1%	62.1±3.2	78.4%	6.8±0.1	133%
7th recycled	2.50±0.07	79.4%	57.6±4.2	72.7%	6.3±0.4	123.5%
8th recycled	2.29±0.08	72.7%	56.1±3.4	70.8%	5.9±0.3	115.7%
9th recycled	2.30±0.05	73.0%	61.8±2.6	78.0%	5.1±0.3	100%
10th recycled	2.20±0.06	69.8%	65.2±5.2	82.3%	4.5±0.1	88.2%

4. Supplementary References

1 Lu, T.-D. *et al.* Electrospun nanofiber substrates that enhance polar solvent separation from organic compounds in thin-film composites. *J. Mater. Chem. A* **6**, 15047-15056 (2018).

2 Wang, D., Yue, Y., Wang, Q., Cheng, W. & Han, G. Preparation of cellulose acetate-polyacrylonitrile composite nanofibers by multi-fluid mixing electrospinning method: Morphology, wettability, and mechanical properties. *Appl. Surf. Sci.* **510**, 145462 (2020).

3 Moazeni, N., Sadrjahani, M., Merati, A. A., Latifi, M. & Rouhani, S. Effect of stimuli-responsive polydiacetylene on the crystallization and mechanical properties of PVDF nanofibers. *Polymer Bulletin* **77**, 5373-5388, (2020).

4 Su, C. *et al.* Dilute solvent welding: A quick and scalable approach for enhancing the mechanical properties and narrowing the pore size distribution of electrospun nanofibrous membrane. *J. Membr. Sci.* **595**, 117548, (2020).

5 Huang, S. *et al.* Preparation and Properties of Electrospun Poly (Vinyl Pyrrolidone)/Cellulose Nanocrystal/Silver Nanoparticle Composite Fibers. *Materials* **9**, 523 (2016).

6 Han, C. *et al.* Enhanced drug delivery, mechanical properties and antimicrobial activities in poly(lactic acid) nanofiber with mesoporous Fe3O4-COOH nanoparticles. *Colloids Surf. A Physicochem. Eng. Asp.* **559**, 104-114 (2018).

7 Wang, X., Fang, D., Yoon, K., Hsiao, B. S. & Chu, B. High performance ultrafiltration composite membranes based on poly(vinyl alcohol) hydrogel coating on crosslinked nanofibrous poly(vinyl alcohol) scaffold. *J. Membr. Sci.* **278**, 261-268 (2006).

8 Cui, J. *et al.* Flexible and transparent composite nanofibre membrane that was fabricated via a "green" electrospinning method for efficient particulate matter 2.5 capture. *J. Colloid Interface Sci.* **582**, 506-514 (2021).

9 Huang, X. *et al.* Flexible PDA@ACNTs decorated polymer nanofiber composite with superhydrophilicity and underwater superoleophobicity for efficient separation of oil-in-water emulsion. *J. Membr. Sci.* **614**, 118500 (2020).

10 Zulfi, A., Hapidin, D. A., Munir, M. M., Iskandar, F. & Khairurrijal, K. The synthesis of nanofiber membranes from acrylonitrile butadiene styrene (ABS) waste using electrospinning for use as air filtration media. *RSC Adv.* **9**, 30741-30751 (2019).
